# Synthetic Organic Compounds From Paper Industry Wastes: Integrated Biotechnological Interventions

**DOI:** 10.3389/fbioe.2020.592939

**Published:** 2021-01-08

**Authors:** Shweta Jaiswal, Guddu Kumar Gupta, Kusum Panchal, Mandeep, Pratyoosh Shukla

**Affiliations:** ^1^Enzyme Technology and Protein Bioinformatics Laboratory, Department of Microbiology, Maharshi Dayanand University, Rohtak, India; ^2^School of Biotechnology, Institute of Science, Banaras Hindu University, Varanasi, India

**Keywords:** synthetic organic compounds, bioremediation, xenobiotics, pollution, pulp and paper industry

## Abstract

Synthetic organic compounds (SOCs) are reported as xenobiotics compounds contaminating the environment from various sources including waste from the pulp and paper industries: Since the demand and production of paper is growing increasingly, the release of paper and pulp industrial waste consisting of SOCs is also increasing the SOCs’ pollution in natural reservoirs to create environmental pollution. In pulp and paper industries, the SOCs *viz*. phenol compounds, furans, dioxins, benzene compounds etc. are produced during bleaching phase of pulp treatment and they are principal components of industrial discharge. This review gives an overview of various biotechnological interventions for paper mill waste effluent management and elimination strategies. Further, the review also gives the insight overview of various ways to restrict SOCs release in natural reservoirs, its limitations and integrated approaches for SOCs bioremediation using engineered microbial approaches. Furthermore, it gives a brief overview of the sustainable remediation of SOCs via genetically modified biological agents, including bioengineering system innovation at industry level before waste discharge.

## Introduction

The paper and pulp industry consumes various raw materials i.e., wood, cellulose-based products, etc. The main aim of the paper and pulp industry is to produce on large scale to figure out the demand. This review insight into the environmental pollution caused by SOCs produced at various processing stages (Table 1). Deforestation for wood has caused a decline in oxygen level worldwide, directly responsible for floods and droughts. Water pollution by waste discharge from pulp industries also contaminates the water bodies with dissolved organic compounds (DOCs), synthetic organic compounds (SOCs), and suspended particles (Gupta and Gupta, 2019; Ramírez-García et al., 2019). The organic compounds reaching humans via water consumption leads to health issues, which are not immediate but show long term effects. The waste discharge also disturbs aquatic life (Karbalaei et al., 2018; Gupta et al., 2019). The emission of harmful chemicals and gases i.e., sulfur dioxide, nitrogen oxide, carbon monoxide will cause acid rain as they are water-soluble and reaches the water bodies indirectly (Gupta and Shukla, 2020). Metlymercaptans, hydrogen sulfides, and dimethyl sulfides along with volatile organic compounds (VOCs) lead to air and water pollution (Singh and Chandra, 2019; Pino-Cortés et al., 2020). The trials for pollution prevention are in continuous use by industries (by use of alternative bleaching agents), environmentalist (by releasing norms) as well as by consumers (by recycling waste and use of sedimentation tanks). Still, these measures are not fulfilling the demand to degrade the SOCs waste from the paper and pulp industry (Zumstein et al., 2018; Liu, 2020). In recent reports, the researchers have shown their interest in the biotechnological advancements for degrading the pollutants (Ellouze and Sayadi, 2016; Tripathi et al., 2017; Sharma et al., 2020). This review covers the advancement in methodologies via engineered biological agents (mainly bacteria) that are reviewed and suggested for sustainable bioremediation of SOCs.

**TABLE 1:** Types of SOCs from the paper industry.

S.no	Synthetic organic pollutant released	Source/Activity/Stage	References
1.	Nitrogen Oxides	Drying process	Zifang et al., 2017; Deviatkin et al., 2019
2.	TRS (i.e., Toxic sulfides)	Fiber paper mill	Sailwal et al., 2020
3.	Carbon Monoxide	Drying process	Mukhametzyanov et al., 2018; Man et al., 2020
4.	Nitrous Oxide	Drying process	Deviatkin et al., 2019
5.	Carbon dioxide	Drying process	Man et al., 2020
6.	VOCs (Volatile Organic Compounds)	Fiber paper mill	Sailwal et al., 2020
7.	Sulfur Oxides	Drying process	Zifang et al., 2017
8.	Dioxins	Pulp effluent	Xiao et al., 2017; Xia et al., 2020
9.	AOX (Adsorbable Organic Halogens)	Pulping stage	Kumar et al., 2020
10.	Furans	Pulp effluent	Hubbe et al., 2016; Romo et al., 2019
11.	Organo siloxane	Washing stage	Zhong et al., 2017; Li and Rabnawaz, 2018
12.	Hydrogen peroxide	Washing stage	Biswas et al., 2019; Elakkiya and Niju, 2020
13.	Mercaptans	Pulping stage	Singh and Chandra, 2019
14.	Sodium hydroxide	Bleaching and washing stage	Yehia et al., 2018; Perzon et al., 2020

## SOCs From Paper Industry Wastes

An ecosystem polluted and damaged by human activities with increasing intensity becomes a primarily global problem. SOCs are of xenobiotic origin in nature and thus there are difficulties involved in biotransformation (Antizar-Ladislao and Galil, 2004; Kumar et al., 2019). Due to recalcitrant, it has ecotoxic effects on the biosphere. SOCs can be primarily produced by following compounds such as methane, ethylene, aliphatics, and aromatics. Among the above, most of the industrial important SOCs derived from the aromatics viz., ethylbenzene, xylene, benzene, and toluene (Fang et al., 2018). Based on their primary uses SOCs are mainly classified such as cyclic, acyclic, aromatics, or aliphatics. SOCs contain huge categories like volatile organics carbons (VOCs), and relatively emerging organic contaminants (EOCs). VOCs primarily contain industrial re-solvents, gasolene agents, trihalomethanes, etc., while EOCs have pharmaceuticals, endocrine disrupting substances, hormones, food additives, microplastics, etc. (Lapworth et al., 2012; Postigo and Barceló, 2015). SOCs are primarily present in wastewater treatment plants. Most of the SOCs pass througfh various photo-transformations or chemical reactions and many of them remain inert in an open environment system.

**FIGURE 1:**
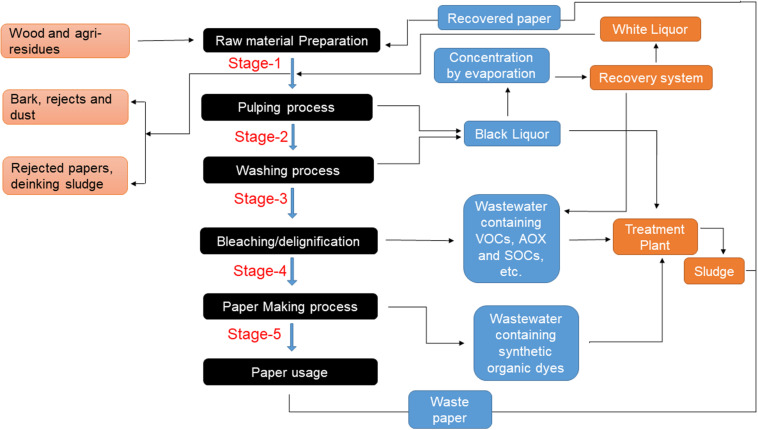
Schematic diagram of pulp processes releasing various SOCs.

In the paper mill, SOCs are released during the pulping and papermaking process. Chlorine and its derivatives have been released and restrained as adsorbable organic halides (AOX) (Savant et al., 2006), while other xenobiotic agents (resin acids, chlorinated lignins, dioxins, phenolic (tannins), and furans) are produced via pulping and paper manufacturing (Chandra et al., 2011). Out of the above, polychlorinated dibenzofurans and dibenzodioxins compounds of furans and dioxins are notably resistant to degradation and are persistent in nature (Gupta and Shukla, 2020). The polar phenolic polymeric compounds (tannins) are released in wastewater during the debarking process of raw wood material, which creates 50% COD of this wastewater (Chandra et al., 2018). Another study revealed that the naturally occurring tricyclic diterpenes (resin acids) are released during the pulping operations, which have pathetic aqua-phobic acids and toxicity levels to aquatic animals at conc. of 200–800 μg/l in wastewater (Duan et al., 2020). Mainly resin acids are produced from the pulping process containing dehydroabietic acid, abietic acid, pimaric acid, isopimaric acid, levopimaric acid, and neoabietic acid (Yadav and Chandra, 2018). Out of all the resin acids, isopimaric acid is notable as highly toxic. Many SOCs are discharged into the water body during the chemical process like calendaring (coating for paper smoothness) in the paper manufacturing industry. The schematic diagram of pulp processes releasing SOCs is given in Figure 1. The dioxins and furans are also released when chlorine reacts with some defoamers and wood preservatives like pentachlorophenol (PCP) during the pulping, washing and pulp bleaching process (Badar and Farooqi, 2012). Additionally, most SOCs that are discharged from the bleaching process areditolyethane, bis (methylphenoxy) ethane, di-iso-propyl naphthalene, terphenyl, chloromethyl-phenoxy-methyl-phenyl-ethane, etc. (Singh and Chandra, 2019). There are a lot of dyes used for paper printing in paper mills. At the end result, approximately 200 billion liters of dye effluents are released based on fabric type and dye used. Many researchers reported that synthetic organic dyes such as azo, phthalocyanine and anthraquinone dyes discharged as effluents in the water body and have the most toxic effect on the environment as well as human health (Tkaczyk et al., 2020).

### Ways to Restrain SOCs Production as Waste

To evaluate and mitigate the hazardous effect and load of SOCs released from the pulp and paper industries into the environment, various processes such as the use of chlorine-free bleaching process, use of ecofriendly chemicals for pulping, use of enzymatic pulping, and bleaching instead of the chemical pulping and bleaching process have been used. Among these, several other advanced and more significant methods have been adopted to reduce the SOCs load into wastewater, which is discussed below.

Many researchers have adopted many significant and ecological important methods help to remove organic pollutants from the environment, viz adsorption, biodegradation, striping, hydrolysis, photolysis, etc. (Ali et al., 2012). But significant results have not yet been obtained. Additionally, conventional adsorption techniques integrated with post-treatment using granular activated carbon (GAC) have been globally adopted for the removal of AOX for pulp mill wastewater. According to Osman et al. (2013), the treatment of paper mill wastewater GAC used with a sequenced batch biofilm reactor (GACSBBR) has significant capability to remove AOX at the longest hydraulic retention time (HRT) (Farooqi and Basheer, 2017). Currently, researchers have revealed that the use of biochar adsorption to mitigate organic pollutants has become an interesting field of research and hotspot. Biochar has a porous structure and contains functional groups of oxygen and minerals (Weber and Quicker, 2018). To eliminate dyes, these dyes go to different types of the treatment process (Puzyn and Mostrag, 2012). The biological, chemical, and physical processes can be done based on wastewater treatment stages (Primary, secondary and tertiary treatment) (Samer, 2015). The removal of organic and inorganic solids takes place in the primary treatment via sedimentation, grinding, and flocculation. While in the biological treatments (secondary treatment), organic materials are used by the aerobic or anaerobic microorganisms by the means of biological oxidation and biosynthesis processes. In the tertiary treatment, the wastewater undergoes different treatment processes like advanced oxidation processes, ion exchange, adsorption and reverse osmosis processes. For example, many researchers used ferric oxide-biochar nanocomposite absorbent extracted from paper mill sludge (Chaukura et al., 2017).

Another study reported/investigated that the biochar can be prepared from cardboard (BCPD), pig manure (BC-PM), and pinewood (BC-PW) for the use in adsorption of various synthetic organic dyes within several pyrolysis terms. Due to high ash content, BC-PM showed significant adsorption properties (Lonappan et al., 2016). Adsorption methods are amongst those used to remove dyes in comparison with other methods (Srivastava et al., 2018). During the degradation process of synthetic organic dyes, it undergoes various transformations kinetics. Some of the changes are into the more toxic agents and some of them non-toxic agents. Advanced techniques such as oxygen cooking techniques, hydrogen peroxide, and ozone treatment for the pulp bleaching process could be options for pretreatment of primary sludge wastes, which helps with the reduction of an environmental load of SOCs production. There are mostly two types of chemical pretreatment used, alkaline and acidic. Acidic pretreatment is promoted for the hemicellulose while alkaline pretreatment for the lignocellulose, which makes it more accessible to use their products (Hendriks and Zeeman, 2009). However, lots of modified methods have been used for the pulping and bleaching process of the pulp mill. Bio-pulping is most suitable for the pulping process using eco-friendly enzymes and it can reduce the production of SOCs in waste materials. Some other techniques like innovation in the bleaching process can be adopted by many researchers. These techniques are elemental chlorine-free (ECF) bleaching techniques and a totally chlorine-free (TCF) bleaching technique (Gupta et al., 2019).

#### Detection and Analysis

Gas chromatography (GC) and Gas chromatography-mass spectrometry (GC/MS) have been used to detect and analyze the SOCs effluent released from the pulp and paper industries. Some metabolites formed by degradation of AOX can be identified by using GC/MS (Pronk et al., 2015). Many researchers used a multi X2500 analyzer to characterize bleaching AOX effluent. A study has stated that organic chlorides were recognized by using GC-MS incorporation with or without hot water abstraction. By these methods, AOXs were categorized into at least four main components such as macromolecular, small molecular organic chloride, chloro-phenol and chlorobenzene. Although, these methods are conventional methods and are time-consuming and expensive. Nowadays, advanced technologies like biosensors have been used, which offer an advantage over classical analytical methods due to their selectivity, sensitivity, eco-friendly, inexpensiveness and short assay time (Yao et al., 2017). However, an immobilized laccase based biosensor has been used for the detection and analysis of organic compounds. Several other electrochemical biosensors such as voltammetric sensor, amperometric laccase biosensor and optical biosensors are used for the analytical analysis of various organic effluents released from industrial operations. Among these, amperometric transducer methods have been reported as widely studied and used in laccase biosensors, while presently optical biosensors have the most significant results in terms of sensitivity (Rodríguez-Delgado et al., 2015). Additionally, a nanomaterial-based (Pena-Pereira et al., 2020) colorimetric detector has been used for the quantitative analysis of low molecular weight gaseous VOCs (Azzouz et al., 2019). Some researchers have employed high-temperature combustion to the transformation of Total organic halides (TOX) into halides and detected and quantified these halides using micro-coulometry methods. In 1977, micro-coulometry titration methods have been replaced by the more reliable ion-selective electrode (ISE) to detect the halides present in the wastes released from the paper mill (Chen et al., 2020).

#### Limitations and Challenges

SOCs such as aromatic compounds (phenols and biphenyls), polycyclic aromatic hydrocarbons (pyrene), are generally discharged into the water bodies. Most of the SOCs found in the environment/wastewater are recalcitrant due to their complexity compared to other effluents. However, these effluents have drawn more attention to treatment systems. These compounds are highly persistent, more toxic compounds that remain over a long period and bio-accumulated into the water body. Separation and treatment of these effluents became mandatory before releasing effluents in the marine ecosystem. For this purpose, the development of efficient techniques has been an interesting area of research for a long time (Awad et al., 2019). The use of conventional technologies has many disadvantages that limit the application area. The main environmental impact is the production of a huge amount of hazardous sludge that creates dumping problems and increases toxicity concentration in treated water (Ashrafi et al., 2013). Traditional methods are more expensive than advanced methods. However, environmental and health costs are also affected by using this classical method. Gaseous emissions, wastewater and sludge production from effluent treatments are relatively unmonitored. In developing countries, these effluents are primarily disposed of into unsecured landfills. The hazardous agents leach out over a long period from the landfills and go directly or indirectly into the environment. Constraints are in place with the purpose of limiting these effects, which have been mandatory across industries (Nimkar, 2017). However, the challenges of the reduction of SOCs production are still under investigation. Researchers have used some innovative and modified technologies for the treatment process of wastewater to help in the mitigation of hazardous compounds in the environment. Mostly SOCs are derived from the aromatic source, viz., toluene, ethylbenzene, anthracene, etc., which are persistent over the period and recalcitrant in the ecosystem because of the rigidness of their molecular structure and present thermodynamically stable aromatic ring (Postigo and Barceló, 2015). The ecotoxic impacts of SOCs on the environment have been accepted and implicit. However, water scarcity, water pollution and water recycling are serious challenges globally (Jain et al., 2020).

### Economical Importance and Hindrance by SOCs for the Paper Industry

Pulp and paper are produced from cellulosic fibers, other plant material and synthetic materials may be used. Papers are mainly derived from wood fibers but cotton liners, bagasse, rags, etc are also used in some papers (Bajpai, 2018). Pulp and paper mills waste material and used papers can be further recycled and used to create economical values. The pulp and paper mills librated a substantial amount of wastewater composed of organic material such as high cellulose, hemicellulose, lignin contents (Kaur et al., 2020). Lignins are cross-linked phenolic polymers. These organic materials are suitable for the derivation of glucose and other fermentable sugars for example galactose, mannose, arabinose, and xylose. By using physical and chemical treatment methods, transformation of paper industry sludge into a glucose-rich liquid can be achieved. Enzymatic hydrolysis is a promising approach for the derivation of sugars from paper industry sludge. Other valuable products can be obtained by causing the fermentation of sugars (Naicker et al., 2020). Production of biofuels such as bioethanol could be successfully achieved by the conversion of pulp and paper industry waste mainly composed of cellulose, hemicellulose, and lignin contents. These components require a series of reaction steps such as hydrolysis, hydrogeoxygenation alkylation, etc to be converted into biofuel. Lignin based biofuels can be produced by using one-pot depolymerization or by the upgrading of bio-oil from biomass decomposition. Pulp and paper industry waste conversion into biofuel is an interesting approach to manage paper industry waste and to create commercial value out of it (Zhu et al., 2020). The paper industry also generated sludge composed of biomass fly ash and calcareous sludge that is commonly disposed of in landfills. Calcareous sludge can be used in the manufacturing of green geopolymeric mortars for the application in construction. These components are released during the Kraft process of lignin. Biomass fly ash was reused as an alternative source of silica and aluminum, and calcareous sludge mainly constituting of calcite, was recycled and used in GP mortars construction. The implemented Mix design was outlined to maximize the incorporation of the calcareous sludge and improve the mortar’s mechanical performance (Saeli et al., 2020). To accomplish a productive re-utilization of waste generated from the paper industry, waste effluent was recycled and used to produce green-composites with high strength which depends on ultra-molecular weight polyethylene, high-density polyethylene, and low-density polyethylene. The three green-composites were developed by an extrusion and injection molding named PLC, PUC, and PHC composites. The maleic anhydride grafted polyethylene, an organic compound, was used as a compatibilizer for preparing composites. The utilization of paper mill waste avoids the environmental waste and also produces the green-composites (Zhang et al., 2020). Anaerobic digestion under mesophilic condition is widely applied for the production of biogas by utilizing waste rich in suspended organic materials liberated from the paper industry. Industry waste contains a very high level of COD and BOD due to the presence of lignin, fatty acids, tannins, resin acids, and chlorinated compounds, etc. This biofilm technology is highly effective in biogas production (Bakraoui et al., 2020). Biogas production can be successfully achieved by using UASB digester technology and it can be applied on a large and small scale. Anaerobic digestion of Recycled pulp and paper industry waste can be carried out at different organic loading rates and in mesophilic conditions (Bakraoui et al., 2020). The amount of lignin is very important in paper manufacturing because lignin will affect the properties of the resultant paper. Lignin amount influences the tensile strength and elongation of cellulose fiber.

### Effect on Ecological and Biological Health

**FIGURE 2:**
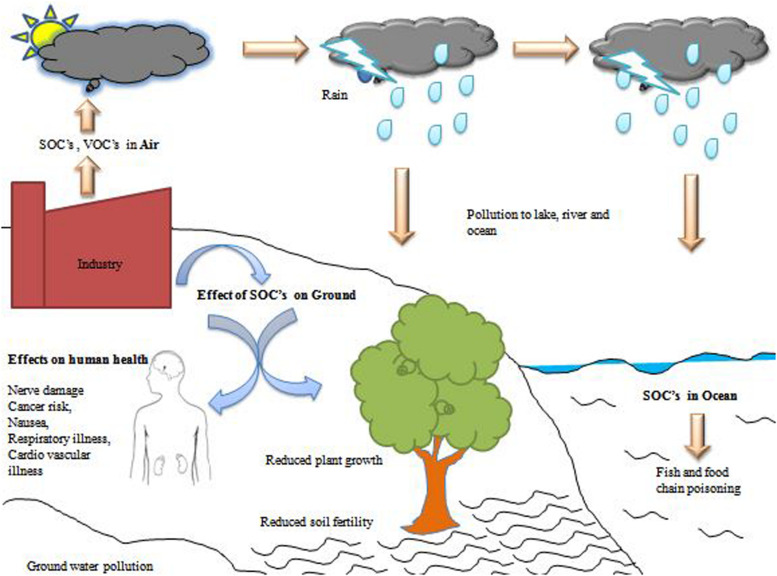
The overall view of SOCs affecting the ecological and biological health.

The production of SOCs comes from mainly the pulping and bleaching stage of the pulp mill. These compounds have toxic properties, which may cause carcinogenic disease, allergic and dermatic disease (Puzyn and Mostrag, 2012). The production of trichlorotrihydroxybenzenes and bromomethylpropanylbenzene in the spent bleach liquor from pulp and paper industries have mutagenicity effects on the aquatic body as well as human beings. Additionally, some other SOCs such as chlorophenols and chloroguaiacols from bleach effluents notably carcinogen, reproductive toxicity in fish, and estrogenic in humans. Further, it has acute toxicity, which prevents the ATP synthesis process and oxidative phosphorylation mechanism (Singh and Chandra, 2019). Some endocrine-disrupting chemicals as residual organic compounds showed chromosomal aberration in marine animals (Chandra et al., 2018). The discharge of black liquor containing SOCs into the environment causes a direct effect on flora and fauna. In a developing country, untreated wastewater released from pulp and paper industries is discharged into the water body (Duan et al., 2020). They have to use this water for irrigation purposes so a lot of hazardous chemicals come into the fields and affected the crops due to changes to the soil properties, like alteration in pH values and beneficial microbes (Nguyen et al., 2020). The organic compounds pass through different trophic levels in the marine ecosystem and are bio-accumulated at a different level, which can be harmful to marine animals. However, the use of biochar for the adsorption of SOCs helps to retain fertilizers in the soil, promoting soil fertility, removal of heavy metals and acids, etc. (Shiralian, 2016). Based on dissipation time, SOCs can be classified into three main categories: highly persistent, moderately persistent, and low persistent. Humans are more exposed to SOCs through polluted air, water, or soil (Bilal and Iqbal, 2019). SOCs combined with their precursors employ eco-toxic effects on the environment (Figure 2; Jaishankar et al., 2014). An experiment was conducted which reported that the effect of SOCs on rainbow trout (*Oncorhynchus mykiss*) in the rivers of Chile, Canada, and Argentina was observed as stimuli for the development of secondary sexual properties and enhanced the intersex features in the young rainbow trout (*Oncorhynchus mykiss*) (Chiang et al., 2015). Similarly, a study conducted in China (2018) reported that long term exposure of andostenrdione has masculinization and reproductive effects in both male and female wastern mosquitofish (*Gambusiaaffinis*) (Hou et al., 2018). Another experiment demonstrated by terasaki and co-workers in 2012 stated that the effects of Dimethyldiphenylmethane and di-iso-propylnaphthalene have reproductive and tissue toxicity on marine fish (Terasaki et al., 2012). The exposure of hexachlorobutadine (HCBD) in human beings has hostile effects on human health either directly or metabolically. The nephrotoxicity effects of HCBD have been observed in animal host experiments and reported as having a necrosis effect on the renal proximal tubule, up-regulation of kidney injuring molecule-1 and lipid peroxidation in renal cells (Sadeghnia et al., 2013). In china, the approximately 8.0 × 10^–6^ μg/kg/day of HCBD exposure dose for human and animal risk was observed which has caused skin diseases, carcinogenicity, sexual abbreviation and mutagenicity in humans as well as aquatic communities (Zhang et al., 2014).

## Biotechnological Interventions in Preserving Environment Through Bioremediation

The recalcitrant nature and toxicological assessment of synthetic organic compounds were not carried out at the early industrial stage. But as the industrialization sector boomed and ill-effects of various pollutants were studied then SOCs also came under scrutiny because of their presence in polluted industrial water. Since then it has become a matter of great concern to remediate these pollutants. Various biological and technological approaches have been utilized to remove SOCs from wastewater before their discharge into water bodies (Jain et al., 2020).

Bioelectrochemical systems, containing electro genesis systems, electro hydro-genesis systems, microbial electrosynthesis (MES) systems (Liu et al., 2018), and microbial desalination systems, are an emerging technology to remediate pollutants (Wang et al., 2015; Fernando et al., 2019). This technology uses electricity and microorganisms to degrade pollutants into less toxic elements. Certain value added products such as biofuels (including hydrogen, butanol, and ethanol, etc.) (Kondaveeti et al., 2019; Liu and Yu, 2020), acetates, and metals are also produced by using these techniques (Moscoviz et al., 2016; Maktabifard et al., 2018). The relatively low energy value (0.2–0.8 V) is needed for the MEC system as compared to conventional water electrolysis (Kadier et al., 2016). Rozendal and co-workers reported that approximately 7 kg COD/m^3^ bioreactor volume/day could be removed by the BES which is the same as a conventional treatment system (Rozendal et al., 2008). Lab scale results reported that MEC showed COD removal efficiency was observed to be about 90–97% of synthetic wastewater at different temperature profile (ranging 5–23°C) and 0.6 kWh/kg electricity. Hence, the BSE is more suitable for small and lab scale systems due to the low energy utilization with improved byproduct production which minimizes the capital cost (Tartakovsky et al., 2018). But the implementation of BES with ordinary systems at industrial levels is more challenging due to the high capital cost which is required (Santoro et al., 2017). Microbial fuel cells (MFCs) are efficient for the biochemical conversion of energy for a useful purpose. Dual-chamber MFC has been utilized for the management of polyaromatic hydrocarbons (PAHs) contagion from diesel. The proposed system detached 82% of PAHs and generated about 31 mW/m^2^ power. MFCs with tubular single- and dual- chambers were applied to reveal *ex situ* and *in situ* management of refinery wastewater or groundwater having a blend of PAHs, containing benzene and phenanthrene (Adelaja et al., 2017). Fenton reaction and the microbial consortium was evaluated for the removal of tannery dye effluent. This exceptional combination was able to remove 89.5% pollutants and led to a reduction in the COD level of 93.7% (Shanmugam et al., 2019). Another advanced oxidation process of ultrafiltration and photoelectrolysis alone was found to remove total phosphorus between 90 and 97% from municipal wastewater and 44% from industrial wastewater (Gray et al., 2020).

Activated carbon has been used as a suitable adsorbent for many pollutants. Superfine powdered activated carbon is found to be more suitable as an adsorbent due to its smaller size, lesser surface oxygen amount, bigger aperture diameters, and neutral pH. An increase in adsorption of planar (phenanthrene) compounds was affected more than non-planar (2-phenyl phenol) compounds (Partlan et al., 2020). Activated carbon can also be used in supporting biofilms for pollutant removal. Due to the larger surface area provided by activated carbon, biomass was able to degrade xylene and other BTEX compounds efficiently and reduce the toxicity of up to 99% (Mello et al., 2019). In this era of machine learning, modeling strategy to check the efficient substrates of adsorption of SOCs can help in the development of efficient adsorbents. In a study, Ghosh et al. (2019) developed a regression support model quantitative structure-property relationship (QSPR). According to this model, they have calculated the adsorption coefficient of 40 SOCs on single-walled carbon nanotubes. They found that various hydrophobic and electrostatic interactions as well as hydrogen bonding help in the adsorption of SOCs on nanotubes. The interaction studies help in the development of suitable adsorbent for SOCs removal from wastewater (Ghosh et al., 2019).

Modified zeolites are also emerging as suitable adsorbents for wastewater treatment. Hashemi et al. (2019) modified a zeolite Y made from bentonite by using CTAB. Various adsorption isotherms indicated removal of 89% total organic carbon and involvement of electrostatic and hydrophobic interactions (Hashemi et al., 2019). Another Fe-nano zeolite was able to absorb phenol (Ph), 2-chlorophenol (2-CP) and 2-nitrophenol (2-NP) in the amount of 138.7, 158.9, and 171.2 mg/g, respectively. This zeolite-based adsorbent was even more cost effective than activated carbon (Tri et al., 2020).

## Sustainable Remediation of SOCs via Genetically Modified Biological Agents

**FIGURE 3:**
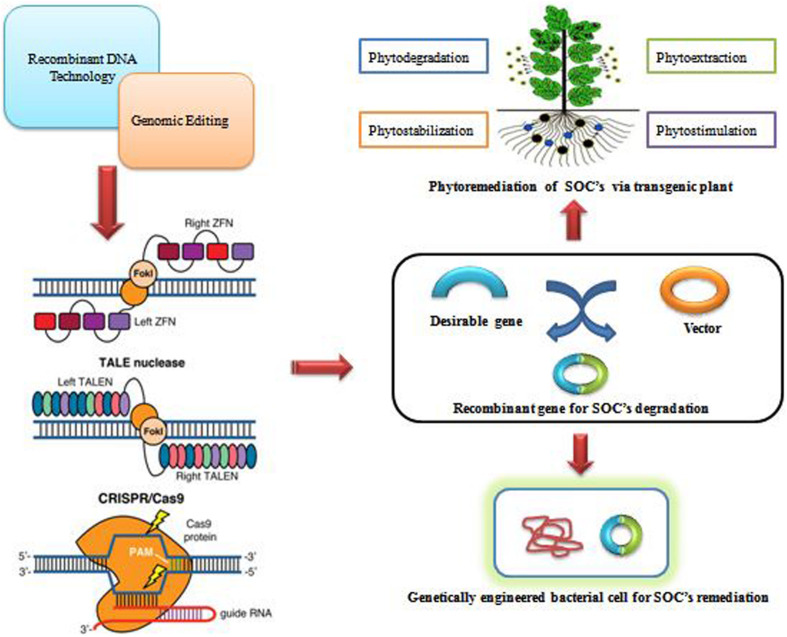
Integrated biotechnological interventions for SOCs pollution treatment.

In the pulp and paper industry paper is derived from wood and produces a huge amount of waste effluents as sludge and polluted water. Toxic chemicals and recalcitrant organic compounds are found in this wastewater (Dixit et al., 2020). Pulp and paper industry waste released into freshwater alters aquatic habitats and adversely impacts human health. The remediation of these organic compounds is necessary to accomplish environmental sustainability. Bioremediation of pollutants is a novel technique to make the effluents less toxic and safe for discarding the waste (Gupta et al., 2019). To protect human lives, the advancement of remediation technologies for the recovery of polluted sites is of utmost importance. Sustainable remediation, which seeks to reduce concentrations to risk-based levels as well as mitigate ancillary environmental consequences such as waste generation, has recently gained significance (Cecchin et al., 2017). Bioremediation requires the use of particular microorganisms to degrade organic pollutants, a reasonable and efficient approach based on microbes’ unique catabolic capacity (Dvořák et al., 2017). This has led to increased efforts using innovative biotechnological methods (Table 2) to develop more effective, ecologically sustainable, environmentally acceptable, and cost-effective remediation technologies (Kumar et al., 2017). Various microorganisms, mainly bacteria and fungi, play an important role to degrade synthetic organic compounds. Degradation of these compounds depends upon the secretion of enzymes by microorganisms that participate in the metabolic pathways. The traditional physicochemical bioremediation methods (*in situ* and *ex situ*) (Jaiswal et al., 2020) are inefficient for degradation and removal of new emerged compounds (Jaiswal and Shukla, 2020). With the development of genetic engineering and Recombinant DNA technology many genetically modified microorganisms were constructed by using various techniques for the remediation of synthetic organic compounds (Liu et al., 2019). Biodegradation of recalcitrant azo dye was successfully done by enzyme azoreductase encoded by gene *azoA* from *Enterococcus* sp. L2 into *E. coli* and *Pseudomonas fluorescens* using the expression vector PBBRMCS2. To further increase the degradation of azo dye NADH regenerate system depended on the formate dehydrogenase enzyme introduced into the host strain by the overexpression of *fdh* gene from *Mycobacterium vacccae* N10. For efficient dye decolorization processes the transcription fusion of *azoA* – *fdh* provided a simple genetic cassette for genetic engineering of an appropriate host (Rathod et al., 2017). Moreover, Biodegradation of phenol and p-nitrophenol was successfully done by genetically modified *Bacillus cereus* strains by introducing the *vgb* gene from *Vitrocilla stercoraria*. The gene was cloned into a pUB110 multicopy plasmid. A higher degradation rate was obtained at 37°C under aerobic conditions by genetically modified bacteria compared with wild type. p-Nitrophenol degradation was obtained high by using the strain with uni-copy of *vgb* gene (Vélez-Lee et al., 2016). *Bacillus cereus* and its recombinant strains are effectively used for biodegradation of phenols and p-nitrophenol under anaerobic and aerobic conditions. Different Phenolic compounds are effectively degraded by the action of Manganese peroxidase, an extracellular heme enzyme of white-rot basidiomycete *Ganoderma*. 1092 bp full-length cDNA of the *MnP* gene, designated *as G. lucidum MnP (GluMnP1)*, was cloned from *G. lucidum* and a eukaryotic expression vector, pAO815: GlMnP was constructed and transferred it into the methylotrophic yeast *Pichia pastoris* SMD116 by the electroporation-mediated transformation. Recombinant GluMnP1 is capable of the degradation of phenol and the degradation of four types of dyes. Great potential for the enzymatic remediation of phenolic compounds and industrial dyes was shown by the Recombinant GluMnP1. Phenol and the main oxidation degradation products including hydroquinone, pyrocatechol, and resorcinol were analyzed by using HPLC (Xu et al., 2017). In another study for the remediation of the phenolic compound engineered *Escherichia coli* effectively used. Nine genes namely, pheA1, pheA2, catA, catB, catC, catD, pcaI, pcaJ, and pcaF were selected from different microorganisms and an oligonucleotide was synthesized. By using the modified overlap-extension PCR method, all synthesized genes were seamlessly connected with the T7 promoter and terminator to construct a gene expression cassette. All the cassettes were transformed to the host *Escherichia coli* strain BL221-AI and the transformant was named BL-phe/cat. The engineered *Escherichia coli* was effectively used for phenol degradation (Wang et al., 2019). Degradation of organophosphates, carbamates, and pyrethroids was achieved by engineering *Pseudomonas putida*. In a study, a scarless genome-editing tool was applied for the engineering of *Pseudomonas putida* KT2440. The vgb and gfp genes were transferred into the chromosome. It was observed that the genetically modified strain *Pseudomonas putida* KTUe having genes (ΔphaC1, Δvdh, ΔalgA/algF, Δfcs, Δupp, ΔphaZ/phaC2, gfp+, mcd+, cehA+, mpd+, pytH+, vgb+) was able to decompose all the pesticides screened. Also, it was found that to sequester oxygen in the soil study with the VHb gene was responsible. Thus, this engineered *Pseudomonas putida* strain is a powerful approach for the degradation of pesticides (Gong et al., 2018). Recent genetic editing technology is a promising approach for engineering the various microorganisms to perform remediation of pollutants (Dangi et al., 2019). With the help of gene-editing techniques, modified microorganisms with maximum quality can be produced by making targeted modifications in the genome using molecular scissors involving engineered nucleases. Clustered regularly interspaced short palindromic repeat (CRISPR-Cas), zinc finger nucleases (ZFNs) and Transcription-activators like effector nucleases (TALENs) are the main gene-editing tools that have the dynamic capacity to boost bioremediation of synthetic pesticides (Jaiswal and Shukla, 2020; Kumari and Chaudhary, 2020). The gene editing process involves self-designed guide sequences that are inserted complementary to the sequence of the gene of interest assisting break at a site, repaired by homologous recombination, insertion, or deletion of desired sequence fragments. A double-stranded (DSB) break can be created by Transcription-activators like effector nucleases in the target sequence on DNA and makes sticky ends. Likewise, zinc finger nucleases also introduce a DSB in the target sequence of the host genome. On another hand, CRISPR-Cas comprise of crRNA and trcRNA joined by gRNA. gRNA controls the Cas9 enzyme to create DSB in the desired sequences of DNA (Jaiswal et al., 2019). In another study plants also play a main role in the removal of various pollutants by phytoremediation. Phytoremediation is a bioremediation form that requires plants as tools for the removal of hazardous contaminants from the environment. Phytostimulation, phytoextraction, phytoextraction, phytostabilization, and phytovolatilization are different approaches of phytoremediation for the remediation of metals/metalloids and other hazardous contaminants. A plant’s genome can be modified by utilizing CRISPR-Cas, ZFNs, and TALENs gene-editing tools (Figure 3; Aminedi et al., 2020). Indeed, clustered regularly interspaced short palindromic repeat (CRISPR-Cas) is a revolutionary genetic engineering tool in plants that provides a pragmatic approach to synthesize advanced phenotypes (Saxena et al., 2020). On another hand, progress in the development of recombinant microorganisms has created potential risks associated with the release into the open environment of such genetically engineered microorganisms (GEMs). But many attempts are being made to monitor and track genetically engineered microorganisms to address these risks. Designing genetically engineered microorganisms by employing sufficient genetic methods to contain the bacterial system will help to reduce the anticipated hazards. For example, transposition vectors are designed which are deemed to be safe in the environment. Another containment technique primarily includes the production of suicidal genetically engineered microorganisms, but the technology has yet to be applied. These advanced technologies are one of the most promising ways to mitigate the adverse effects of genetically engineered microorganisms release in the open environment (Hussain et al., 2018). But certain risks could also exist and further study will then be needed to produce acceptable technical regulatory guidelines.

**TABLE 2:** Advance biotechnological techniques for SOCs level reduction.

S.no	Approach	Methodology	Model organism used	Application	References
1.	Biotechnological approach	GMOs	Bacteria	*Pseudomonas putida*	Tahseen et al., 2019; Lindemann et al., 2020; Yang et al., 2020
				*Klebsiella* spp.	Shang et al., 2019; Rhie et al., 2019
				*Achromobacte*r	Yang et al., 2020
2.	Synthetic biology	Gene editing tools	Crispr-Cas	Gene-specific nucleases	Stein et al., 2018
			TALEN		Jaiswal et al., 2019
			Zn Finger		Kumar N.M. et al., 2018; Kumar V. et al., 2018
3.	Systems biology	Biodegradation network	BioCyc	Bioremediation	Jaiswal et al., 2019
				Biotransformation	
		Metabolic network	MetaCyc	Xenobiotics metabolism;	Jassal et al., 2020
				Dynamics study;	
				Genome-scale metabolic database	
		Enzymatic reactions	KEGG	Enzymes encoding genes;	Kanehisa, 2017; Li et al., 2018
				Metabolic enzymes	
		Omics	Genomic; metagenomics; High throughput analysis; Proteomics		Malla et al., 2018; Gupta et al., 2020

## Conclusion and Future Perspective

The review shows the extent that the recent research in the field of environmental pollution by the paper and pulp industry has reached. The researchers and environmentalists concluded that SOCs pollutant levels must be declined, and have worked in the same direction. They found that the composition of various chemicals varies with the stage and methodologies applied for paper production. The detection and degradation of organic chemicals produced during paper production are enhanced by researchers using advanced techniques. Biotechnological intervention using synthetic and systems biology for producing genetically modified organisms specifically for potential degradation of SOCs came into consideration. Thus, this review covers the recent reports and methodologies used by the researcher for environmental sustainability.

## Author Contributions

SJ wrote the first draft of the manuscript with contributions from GK, M, and KP. PS read and edited the final draft. All authors approved the final draft for its submission.

## Conflict of Interest

The authors declare that the research was conducted in the absence of any commercial or financial relationships that could be construed as a potential conflict of interest.
